# Studying a collection of common-wheat varieties for leaf rust resistance, crop yield and grain quality in the environmental conditions of Novosibirsk region

**DOI:** 10.18699/VJGB-23-114

**Published:** 2023-12

**Authors:** L.P. Sochalova, V.A. Aparina, N.I. Boyko, E.V. Zuev, E.V. Morozova, K.K. Musinov, N.A. Vinichenko, I.N. Leonova, V.V. Piskarev

**Affiliations:** Siberian Research Institute of Plant Production and Breeding – Branch of the Institute of Cytology and Genetics of the Siberian Branch of the Russian Academy of Sciences, Krasnoobsk, Novosibirsk region, Russia; Siberian Research Institute of Plant Production and Breeding – Branch of the Institute of Cytology and Genetics of the Siberian Branch of the Russian Academy of Sciences, Krasnoobsk, Novosibirsk region, Russia; Siberian Research Institute of Plant Production and Breeding – Branch of the Institute of Cytology and Genetics of the Siberian Branch of the Russian Academy of Sciences, Krasnoobsk, Novosibirsk region, Russia; Federal Research Center the N.I. Vavilov All-Russian Institute of Plant Genetic Resources (VIR), St. Petersburg, Russia; Siberian Research Institute of Plant Production and Breeding – Branch of the Institute of Cytology and Genetics of the Siberian Branch of the Russian Academy of Sciences, Krasnoobsk, Novosibirsk region, Russia; Siberian Research Institute of Plant Production and Breeding – Branch of the Institute of Cytology and Genetics of the Siberian Branch of the Russian Academy of Sciences, Krasnoobsk, Novosibirsk region, Russia; Institute of Cytology and Genetics of the Siberian Branch of the Russian Academy of Sciences, Novosibirsk, Russia; Institute of Cytology and Genetics of the Siberian Branch of the Russian Academy of Sciences, Novosibirsk, Russia Novosibirsk State Agrarian University, Novosibirsk, Russia; Siberian Research Institute of Plant Production and Breeding – Branch of the Institute of Cytology and Genetics of the Siberian Branch of the Russian Academy of Sciences, Krasnoobsk, Novosibirsk region, Russia

**Keywords:** common wheat, leaf rust, population, isolate, virulence, resistance gene, yield, microelement, macroelement, protein, gluten, пшеница мягкая, бурая ржавчина, популяция, изолят, вирулентность, ген устойчивости, урожайность, микроэлемент, макроэлемент, белок, клейковина

## Abstract

The relationship between a variety’s genotype, environmental conditions and phytopathogenic load are the key factors contributing to high yields that should be taken into account in selecting donors for resistance and high manifestation of valuable traits. The study of leaf rust resistance in 49 common wheat varieties was carried out in the field against the natural pathogen background and under laboratory conditions using single-pustule isolates with virulence to Lr9 and Lr24. It has been shown that the varieties carrying alien genes Lr6Agi2 (Tulaikovskaya 10) and Lr6Agi1 (Voevoda) were resistant to leaf rust infection both in the field and in the laboratory. Varieties KWS Buran, KWS Akvilon, KW 240-3-13, and Etyud producing crop yields from 417 to 514 g/m2 comparable to the best standard variety Sibirskaya 17 can be reasonably used as Lr24 resistance gene donors under West Siberian conditions. Omskaya 44 variety showing crop yield of 440g/m2 can be used as a donor for Lr19 and partially effective Lr26. Varieties Tuleevskaya and Altayskaya 110 with Lr9 in their genomes are recommended for the development of resistance gene-pyramided genotypes. The highest protein and gluten contents were observed in the CS2A/2M sample, while KWS Buran, Altayskaya 110, Volgouralskaya, and KWS Akvilon showed the lowest values. Varieties CS2A/2M, Tulaikovskaya 10, Pavon, and Tuleevskaya were ranked the highest in micro- (Cu, Mn, Zn, Fe) and macronutrient (Ca, Mg, K) contents among the common wheat samples from the collection, while the lowest values for most elements were observed in KWS Buran, Novosibirskaya 15, and Volgouralskaya. Winter varieties demonstrating leaf rust resistance against the infectious background typically carry adult plant resistance genes (Lr34, Lr12, and Lr13), particularly combined with the juvenile Lr26 gene. The presence of Lr41 in a winter type line (KS 93 U 62) allowed it to maintain resistance against a leaf rust pathogen clone kLr24, despite the presence of Lr24 in the genotype. Varieties Doka and Cheshskaya 17 may act as donors of resistance genes Lr26 + Lr34 and Lr9 + Lr12 + Lr13 + Lr34, as well as sources of dwarfing without losses in winter hardiness and yield under West Siberian conditions

## Abstract (ru)

К основным факторам, влияющим на формирование высокого урожая, относятся связь генотипа сорта с условиями произрастания и фитопатогенная нагрузка, что необходимо учитывать в селекции для поиска доноров устойчивости и высокой выраженности ценных признаков. Изучение устойчивости 49 образцов мягкой пшеницы к поражению бурой ржавчиной проведено в полевых условиях естественного инфекционного фона и в лабораторных условиях к монопустульным изолятам с вирулентностью к генам Lr9 и Lr24. Показано, что сорта, несущие чужеродные гены Lr6Agi2 (Тулайковская 10) и Lr6Agi1 (Воевода), устойчивы к поражению бурой ржавчиной как в полевых условиях, так и при заражении в лаборатории. Сорта KWS Buran, KWS Akvilon, KW 240-3-13 и Этюд, которые формировали урожайность от 417 до 514 г/м2 – на уровне лучшего стандарта Сибирской 17, целесообразно использовать в условиях Западной Сибири в качестве доноров гена устойчивости Lr24. Донором генов устойчивости Lr19 и частично эффективного Lr26 может служить сорт Омская 44, характеризующийся урожайностью 440 г/м2. Сорта Тулеевская и Алтайская 110, в геноме которых содержится ген Lr9, рекомендуется использовать при создании генотипов с пирамидой генов устойчивости. Наиболее высокие показатели содержания белка и клейковины выявлены у образца CS2A/2M, наименьшие – у сортообразцов KWS Buran, Алтайская 110, Волгоуральская и KWS Akvilon. Сравнение коллекции образцов мягкой пшеницы по микро(Cu, Mn, Zn, Fe) и макроэлементам (Ca, Mg, K) продемонстрировало наиболее высокие показатели у группы, состоящей из образцов CS2A/2M, Тулайковская 10, Pavon и Тулеевская. Наименьшие показатели большинства элементов определены у сортов KWS Buran, Новосибирская 15 и Волгоуральская. Озимые сорта, характеризующиеся устойчивостью к поражению бурой ржавчиной в условиях инфекционного фона, как правило, несут возрастные гены устойчивости (Lr34, Lr12 и Lr13), в том числе в сочетании с ювенильным геном Lr26. У линии с озимым типом развития (KS 93 U 62) выявлен ген Lr41, благодаря чему линия сохраняла устойчивость к поражению клоном патогена бурой ржавчины кLr24, несмотря на наличие в ее генотипе гена Lr24. Сорта Дока и Чешская 17 могут быть донорами генов устойчивости Lr26 + Lr34 и Lr9 + Lr12 + Lr13 + Lr34 и источниками короткостебельности без снижения зимостойкости и урожайности в условиях Западной Сибири.

## Introduction

Common wheat (Triticum aestivum L.) is recognized as the primary food crop around the world. It is characterized by balanced composition of protein, starch, fiber, fat, and mineral elements, while also including vitamins С, В, А, E, D, K, beta carotene etc. (Roshan et al., 2016) and demonstrating high adaptability to growing conditions (Prya-nishnikov, 2018). According to the data for 2022 (Rosstat), the area under crops for spring wheat varieties in the Novosibirsk region was 222,808 ha with crop yield of 21 centner/ha. The area for winter varieties was about 34,000 ha with crop yield of 28 centner/ha. The key factors contributing to high crop yields are the relationship between a variety’s genotype, its growing conditions (Malchikov, Myasnikova, 2012) and phytopathogenic background. Developing a high-yielding variety requires taking these factors into account while selecting donors for resistance and high intensity of valuable agronomic traits (Volkova et al., 2016).

Wheat leaf rust is among the most common diseases found in bread wheat in West Siberia, as it affects both winter and spring varieties and reduces crop yields by 15–40 % in epiphytotic years (Kolmer et al., 2015). There is a set of requirements applying to developing and handling resistance gene donors, since the use of identical genes in spring and winter varieties may lead to an epiphytotic outbreak, if the pathogen overcomes the defenses ensured by the gene (Volkova et al., 2016; Pozherukova et al., 2019). Thus, winter and spring varieties require different effective resistance genes and their combinations for protection against the infection, which implies continuous research efforts to find new resistance genes.

Over 80 Lr genes have been identified around the world, with about 50 % classified as alien ones. Komugi – wheat genetic resources database. Available: https://shigen.nig.ac. jp/wheat/komugi/genes/symbolClassListAction.do?geneClassificationId=89 (accessed on March 9, 2023). The list of genes used in commercial common wheat varieties includes Lr9, Lr19, Lr21, Lr23, Lr24, Lr26, Lr28, Lr37, Lr39 (Aktar-Uz-Zaman et al., 2017; Leonova, 2018), Lr6Agi1, Lr6Agi2 (Sibikeev et al., 2017), and LrSp2 (Adonina et al., 2018). In Russia, breeding value is assigned to the samples carrying partially effective genes Lr9, Lr19, Lr24, Lr25, Lr26, Lr6Agi1, Lr6Agi2 and highly effective protective genes Lr28, Lr29, Lr39, Lr42, Lr45, Lr47, Lr50, Lr51, Lr66, LrSp2 (Gultyaeva, Shaydayuk, 2021; Sochalova et al., 2022).

The use of wheat varieties carrying resistance genes from relative species (Aegilops, Agropyron, Secale cereale, etc.) for hybridization makes it possible to extend the diversity of resistance genes, although the latter are often linked to the factors reducing crop yields or quality (Markelova, 2007; Krupin et al., 2019). It was found that the presence of a fragment carrying Lr9 (transferred from Aegilops umbellulata) reduced crop yield in the United States (Friebe et al., 1996), while commercial varieties carrying this gene are available in Russia (Gultyaeva, Shaydayuk, 2021). The presence of alien material (gene Y determining an increase in yellow pigment synthesis in endosperm) linked to gene Lr19 (transferred from Agropyron elongatum) reduced the value of the first donors carrying this gene (Knott, 1968). Later, the locus carrying Lr19/Sr25 was successfully separated from gene Y using ph1b deletion lines (Marais, 1992; Zhang et al., 2005). A chromosome segment carrying Lr19 was shown to have a positive effect on crop yield (Singh et al., 1998), and a number of varieties with this gene are currently in production in Russia (Gultyaeva, Shaydayuk, 2021). The presence of a fragment carrying Lr38 (transferred from Thinopyrum intermedium) in the wheat genome causes a significant reduction in crop yield (Mebrate et al., 2008) similarly to the presence of a chromosome segment including Lr47 (transferred from Aegilops speltoides), which on top of that has a negative effect on quality (Brevis et al., 2008). Introduction of a wheat-rye transloca- tion 1В.1R carrying genes Lr26, Pm8, Sr31 leads to deteriorating quality of flour and bread (Kumlay et al., 2003). The use of currently available common wheat lines and varieties with alien translocations makes breeding efforts significantly easier, as it does not require obtaining new breeding material with the primary transfer from relative species (Timonova et al., 2012). Direct hybridization are not always successful, and translocations may be partially lost in the offspring upon further reproduction (Davoyan et al., 2015).

Among other things, selection of pairs for crosses is guided by environmental and geographic differences, which is explained by the high diversity of the genotypes obtained as a result of transgressions in segregating generations in crosses between varieties intended for and adapted to different conditions (Vyushkov, 2004). However, the adaptability of alien samples to local conditions is to be taken into account (Davydova, Kazachenko, 2013), because the use of environmentally distant samples with low adaptability produces a significant number of low-yielding genotypes in the offspring, which complicates the development of commercial varieties (Souza, Sorrells, 1991). The use of landraces as donors is complicated by the lack of research and their heterogeneity, since they were created as populations and have multiline nature. Thus, modern varieties of Russian and foreign breeds appear to be the best source for breeding, but a comprehensive investigation of their behavior under local conditions is required beforehand.

We suppose that selection of wheat leaf rust resistance donors is relevant in a close connection with target soil and climatic conditions, as well as with type of development. Therefore, the goal of the present paper was to perform a comprehensive investigation of the collection of common wheat varieties in the Novosibirsk region to identify donors of effective resistance genes for Puccinia triticina Erikss.

## Materials and methods

In the present paper, we studied a collection common-wheat samples including 24 spring varieties and 25 winter varieties, among which 41 samples were from the VIR global collection and eight new spring varieties had been recently tested in Novosibirsk branch of the State commission of the Russian Federation for selection achievements test and protection (FSBI “GOSSORTCOMMISSION”).

The field resistance to local population of leaf-rust pathogens was studied against the natural spread of the infection according to the VIR methodology (Merezhko et al., 1999) and against the artificially increased infectious background (sowing of susceptible winter wheat varieties, spraying the seedlings early in the morning with water and urediniospore mixture upon the emergence of the disease). Crop yield and its components (1000 grain weight, grain weight per spike, grain number per spike) was evaluated in the samples for 2–4 years (within the 2015–2020 evaluation of samples in collection nurseries) according to the VIR methodology developed for new acquisitions (Merezhko et al., 1999). The leaf rust resistance at juvenile stage was studied under laboratory conditions at the Siberian Research Institute of Plant Production and Breeding (SibNIIRS, Krasnoobsk, Novosibirsk region) in leaf fragments (Mikhailova, Kvitko, 1979). The samples were inoculated with water suspension of urediniospores prepared from the local population of P. triticina collected in 2020 from wheat varieties cultivated under natural conditions in the SibNIIRS fields (virulence for varieties and lines with genes Lr1, Lr2a, Lr2c, Lr3a, Lr9, Lr16, Lr3ka, Lr11, Lr17, Lr30, Lr2b, Lr3bg, Lr14a, Lr14b, Lr15, Lr18, Lr20; avirulence to Lr24, Lr19, Lr41, Lr45, Lr47, Lr28, Lr6Agi1, Lr6Agi2, LrSp2, and Lr26) and two testing clones: кLr24 (virulence to Lr1, Lr2a, Lr2c, Lr3, Lr3ka, Lr11, Lr24, Lr17, Lr30, Lr2b, Lr3bg, Lr14a, Lr14b, Lr15, Lr18, Lr20; avirulence to Lr9, Lr16, Lr26, Lr19) and kLr9 (virulence to Lr1, Lr2a, Lr2c, Lr3, Lr9, Lr16, Lr3ka, Lr11, Lr17, Lr30, Lr2b, Lr3bg, Lr14a, Lr14b, Lr15, Lr18, Lr20; avirulence to Lr24, Lr26, Lr19). A clone with virulence to p24 was isolated from variety Novosibirskaya 15 during the study of race composition of the population from the Kuibyshev District of the Novosibirsk region. A clone with virulence to p9 was isolated from variety Chelyaba 2 (Lr9) cultivated in the collection nursery in the settlement of Krasnoobsk. Agent (with Lr24) and Udacha (with Lr9) were used as control varieties. Infection response type (IT) was determined on the 8–10th day after inoculation using the scale proposed by E.B. Mains and H.S. Jackson (1926), with 0, 1, 2 representing resistant response; 3, 4 susceptible response, and Х heterogeneous response (Mains, Jackson, 1926). Virulence of the population and clones was determined in isogenic Thatcher lines and varieties carrying the known resistance genes. The severity of the damage done to the varieties in presence of artificial infectious background was estimated according to the quantitative scale proposed by R.F. Peterson et al. (1948). Novosibirskaya 15 variety was used as a susceptible control in the field and in the laboratory.

Total DNA was isolated from 5–7-day seedlings using the method proposed by J. Plaschke et al. (1995). Genotyping of wheat varieties was performed using the DNA markers developed for wheat leaf rust resistance genes (Supplementary Material 12). Protein and gluten contents were measured using an OmegAnalyzer G near-infrared spectrometer (Bruins Instruments, Germany). Macro- and micronutrient contents were measured using a ContrAA 800 D atomic absorption spectroscope (Analytik Jena, Germany).


Supplementary Materials are available in the online version of the paper: https://vavilov.elpub.ru/jour/manager/files/Suppl_Sochalova_Engl_27_8.pdf


Statistical processing of the results was performed using Statistica 10.0 and MS Excel.

## Results

Evaluation of leaf rust resistance of the tested wheat varieties against the 2020 pathogen background has enabled us to identify 20 spring and 21 winter varieties with disease severity rates of 10 % and below (Table 1). The severity rate in Zauralochka, Udacha, Altayskaya 110, and Tuleevskaya varieties carrying Lr9 gene reached up to 100 % of the susceptibility standard level (Novosibirskaya 15 variety) under field conditions. At the same time, almost all varieties were ranked moderately resistant (score 5) against the natural spread of the infection in the years with maximum pathogen background (Table 2). Juvenile resistance to P. triticina was maintained in 20 spring wheat varieties and only 10 winter varieties (Amigo, KS 93 U 50, KS 90 WGRC 10, KS 93 U 40, KS 93 U 62, Poema, Aivina, Kollega, Pervitsa, Vostorg), which implies the presence of adult plant resistance genes in the remaining 11 winter varieties (Knyaginya Olga, Doka, Lebed, Kuma, Batko, Grom, Lidiya, CO 07 W 245, Ritter, Cheshskaya 16, and Cheshskaya 17 (see Table 1).

**Table 1.**
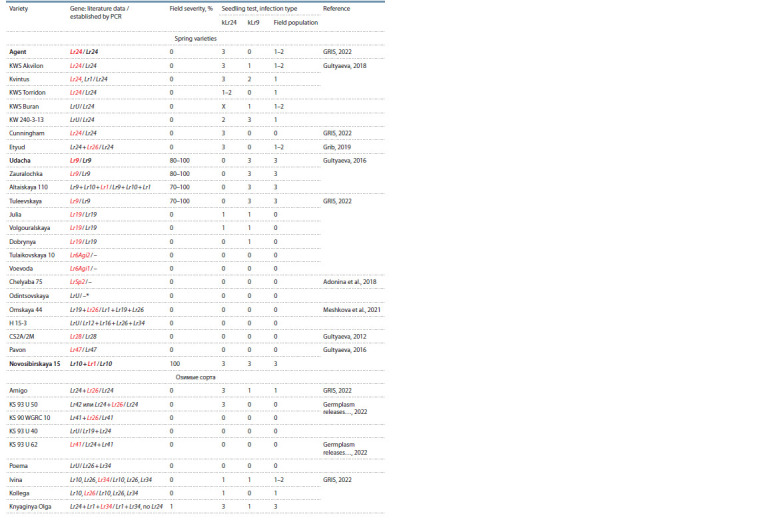
Evaluation of disease infection rate in common wheat varieties with established Lr resistance genes

**Table 1.end:**
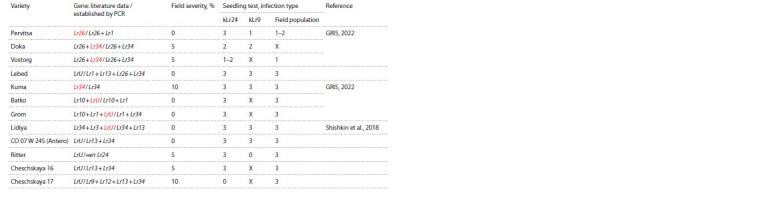
Table 1.end Notе. Resistance genes presented according to literature data are highlighted in red. * According to the pedigree, the presence of the LrSp2 gene is assumed, a dash (–) means that the identification of the Lr gene using the PCR method was not performed.

**Table 2.**
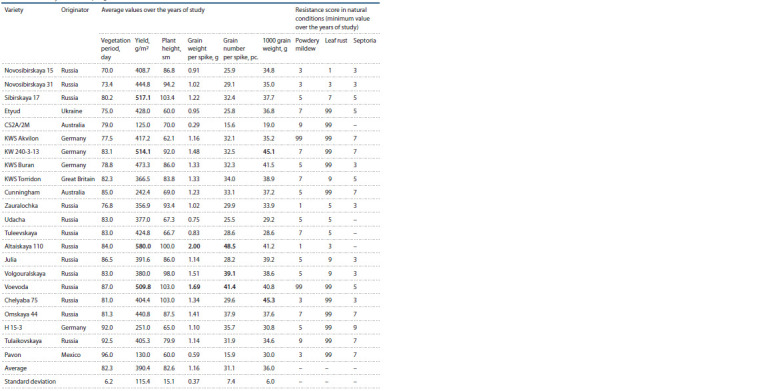
Field study results for spring common wheat varieties Septoria leaf spot resistance was not evaluated in the years when the sample was studied.

The results of molecular testing using markers developed for resistance genes Lr1, Lr9, Lr10, Lr12, Lr13, Lr16, Lr19, Lr24, Lr26, Lr28, Lr34, Lr41, and Lr47 confirmed the presence of postulated Lr genes in most varieties being studied. In addition, we found that KWS Buran and KW 240-3- 13 varieties studied in collection nurseries carried Lr24 gene similarly to the other modern varieties from the EU (KWS Akvilon and KWS Torridon). It is worth noting that KW 240-3-13 was infected by the кLr9 clone, but resisted the kLr24 clone (IT 2, i. e. moderately resistant), while KWS Buran showed heterogeneous response to the kLr24 clone and resisted the kLr9 clone.

Saratov breed varieties Tulaikovskaya 10 and Voevoda carrying alien genes Lr6Agi2 and Lr6Agi1 maintained resistance to pathogen in the field (in particular, against the artificial pathogen background) and to the clone with virulence to Lr24. We were unable to find any publicly available information on wheat leaf rust resistance genes carried by the H 15-3 variety, which demonstrated leaf rust immunity against pathogen in field and in the laboratory testing. Based on the genotyping results obtained using molecular DNA markers, resistance genes Lr12 + Lr16 + Lr26 + Lr34 were found.

The presence of Lr41 gene detected in the genomes of the winter lines developed at the University of Kansas (USA) (KS 90 WGRC 10 and KS 93 U 62) allowed the line KS 93 U 62 maintain resistance to the кLr24 clone, despite the presence of the Lr24 gene. The KS 93 U 40 line characterized by the presence of two Lr genes (Lr19 + Lr24) also maintained resistance to the kLr24 clone as the spring varieties carrying Lr19 (Yuliya, Volgouralskaya, Dobrynya). At the same time, according to the literature, the KS 93 U 50 line carrying the Lr26 and Lr24 genes was susceptible to the kLr24 clone, but maintained resistance to both the native population and the kLr9 clone in the context of increased infectious background. The Lr19 and Lr26 genes in the genotype of the Omskaya 44 spring variety effectively protected the plants from both the native population of wheat leaf rust pathogen and clones.

Noteworthy results were obtained for winter varieties characterized with different combinations of resistance genes, e. g., adult plant resistance gene Lr34 combined with juvenile resistance gene Lr26 in the Kollega, Poema, Aivina, and Doka varieties allowed them to maintain resistance both against natural pathogen background and pathogen clones (see Table 1). At the same time, the Lebed variety carrying Lr13 (adult plant resistance gene) in addition to Lr26 and Lr24 genes was affected both by the native population and the clones in the juvenile phase and overcame infection in the field against the increased infectious background. A similar response was observed in Lidiya, Cheshskaya 16, and CO 07 W 245 varieties with two adult plant resistance genes (Lr13 + Lr34) identified in the genome.

Effective use of resistance donors implies their fitness to the local conditions, which is why we analyzed the crop yields and manifestation of quantitative traits in a number of varieties tested in various experiments in different years. The selected varieties and lines had been under study for at least two years. Based on field evaluation of valuable agronomic traits, the following high-yielding spring wheat varieties stood out: Voevoda (509.8 g/m2), KW 240-3- 13 (514.1 g/m2), and Altayskaya 110 (580.0 g/ m2) (see Table 2). On top of that, Voevoda and Altayskaya 110 produced high-yielding spikes (1.69 and 2.00 g) with high number of grains per spike (41.4 and 48.5). The KW 240- 3-13 variety produced large grains with high 1000-grain weight (45.1 g). Other results of note included the Volgouralskaya (with high ear grain content of 39.1) and Chelyaba 75 (with 1000 grain weight of 45.3 g) varieties. In context of intensive crop farming practices, special attention is paid to dwarf varieties. Etyud, KWS Akvilon, KWS Torridon, and Tulaikovskaya 10 were not only resistant to the pathogen, but also showed crop yields comparable to the best standard variety Sibirskaya 17 (517.1 g/m2) while being short-stemmed (62.1–83.8 cm) and so can be recommended as a source for developing leaf rust resistant varieties for intensive crop farming. Etyud, KWS Akvilon, KW 240-3-13, Omskaya 44, and Tulaikovskaya 10 were characterized by high resistance (scores 7–99) to powdery mildew and septoria leaf spot during the years with high pathogen activity, high resistance to septoria leaf spot alone was observed in Н 15-3 (score 9), Cunningham, and Pavon (7) varieties, while Voevoda, Tuleevskaya, and KWS Torridon were resistant to powdery mildew (7), which is also a significant trait for selecting pairs for crosses.

Among the leaf rust resistant winter varieties, high crop yields were demonstrated by Doka (589.2 g/m2) and Cheshskaya 17 (547.7 g/m2) also characterized by short stems (66.5 and 80.0 cm respectively) and winter hardiness comparable to standard variety Novosibirskaya 40 (score 4.1) (Table 3). In addition, Doka variety produced high number of grains per spike (100.6).

**Table 3.**
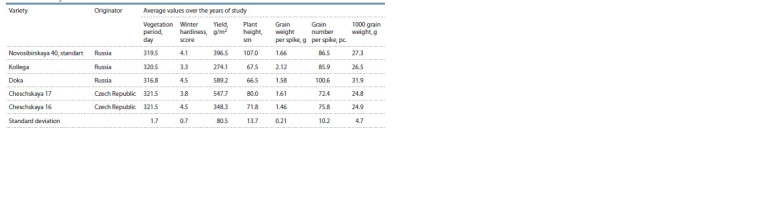
Field study results for winter common wheat varieties Protein (a) and gluten (b) contents in grains of spring common wheat varieties.

Protein and gluten content varied from 13.4 to 22.95 % and from 25.94 to 46.33 % respectively, with CS2A/2M demonstrating significantly higher values compared to other varieties ( p < 0.001) (the Figure, Supplementary Material 2). KWS Buran, Altayskaya 110, Volgouralskaya, and KWS Akvilon varieties were characterized by the lowest protein contents below 14 %. The lowest gluten content values were observed in Volgouralskaya, KWS Akvilon, and KWS Buran varieties

Comparison of micro- (Cu, Mn, Zn, Fe) and macronutrient (Ca, Mg, K) contents in the studied varieties showed that the highest values were observed in the CS2A/2M, Tulaikovskaya 10, Pavon, and Tuleevskaya varieties. The lowest values for most elements were observed in the KWS Buran, Novosibirskaya 15, and Volgouralskaya varieties.

## Discussion

Despite the significant advances in biotechnology, hybridization of initial parental forms with further selection of morphotypes of interest (Gultyaeva et al., 2020; Marchenko et al., 2020), in particular, using marker-assisted selection (Stasyuk et al., 2017; Gultyaeva et al., 2018), still remains the prevalent method of developing new wheat varieties. Leaf rust is among the most dangerous diseases of wheat in West Siberia, as it affects both winter and spring varieties. To prevent epiphytotic outbreaks accompanied by dramatic reductions in crop yields of spring varieties, plant breeders have to use different effective resistance genes and their combinations for winter and spring varieties (Krupin et al., 2019). Another significant factor in selecting resistance donors is their fitness to target conditions, because resistance gene donors are often represented by foreign breeds (Gryaznov, Pigorev, 2019; Konkova et al., 2022) or the isogenic lines developed based on foreign cultivars (Koishybaev, 2019), and these genotypes can show reduced crop yields under local conditions due to low adaptability to adverse abiotic environmental stresses. In the present study, we have performed a comprehensive evaluation of wheat leaf rust resistant varieties. So, among the spring varieties carrying Lr24 gene, German breed varieties (KWS Buran, KWS Akvilon), English KWS Torridon variety, or Ukrainian Etyud variety can be reasonably used as resistance donors for West Siberian conditions, unlike the Australian variety Cunningham producing much lower crop yield (242.4 g/m2) compared to the minimum crop yield of a standard variety of 408.7 g/m2 (Novosibirskaya 15). The latter drop in crop yield has nothing to do with the alien translocation from Thinopyrum elongatum (Lr24/Sr24), but is rather due to low adaptability of the genotype as a whole, which may have a detrimental effect on selection of high-yielding forms, if Cunningham variety is used as a donor for Lr24 gene.

The Lr19 gene commonly used in Russian breed varieties (Gultyaeva, Shaydayuk, 2021) still remains rather effective in protecting wheat varieties from leaf rust infection in West and East Siberia (Gultyaeva et al., 2018; Meshkova et al., 2019), despite its defense being compromised in the European part of Russia (Gultyaeva et al., 2020). The Omskaya 44 variety (440.8) can act as a donor for this resistance gene, since it is comparable to the best standard variety Sibirskaya 17 in crop yield (517.1 g/m2). Apart from that, this variety can also act as a donor for the Lr26 gene, which is partially effective in protecting wheats from leaf rust in West (Gultyaeva et al., 2018) and East Siberia (Meshkova et al., 2019).

Despite the failure to resist infection in the context of infectious background, varieties carrying the Lr9 gene still have breeding value, since it protects plants from severe infection in context of natural infection spread. Varieties carrying this gene (except for Altayskaya 110) were ranked as moderately resistant to the pathogen during the years of its maximum activity. The Lr9 gene donors may be used for developing resistance gene-pyramided varieties, which may prolong the lifespan of the gene.

Breeding value of the donors of the Lr28 (CS2A/2M) and Lr47 (Pavon) resistance genes transferred from Aegilops speltoides seems questionable under West Siberian conditions, since their low fitness to the local conditions drastically affects the crop yields (125.0 and 130.0 g/m2 respectively). On top of that, the evaluation of breeding material collected from hybrid populations F3 and BC1F3 obtained earlier on the basis of two commercial varieties (Sibirskaya 17 and Novosibirskaya 31) crossed with lines Thatcher Lr28 and Thatcher Lr47 (Piskarev et al., 2021) showed a significant increase in vegetation period (+ 6.3 days) compared to the recipient Sibirskaya 17 (44.2 days) and in plant height (+ 11.4 cm) in recombinants with the Lr28 gene. Adverse effects on crop yield, number of grains per spike, and stem length were observed in recombinants with Novosibirskaya 31 variety carrying the Lr47 gene.

Despite the relatively high crop yield of the Chelyaba 75 variety (404.4 g/m2) carrying the LrSp2 gene from Aegilops speltoides Tausch linked to the gametocidal gene (Adonina et al., 2018), which is surely a valuable trait under West Siberian conditions, we were unable to obtain a variety outperforming the current standards while carrying this gene, despite the availability of vast source material (over 4000 lines from crosses between four varieties, namely Novosibirskaya 15, Novosibirskaya 31, Udacha, and Sibirskaya 17) as early as 2015.

Wheat leaf rust resistance of the Odintsovskaya variety (selection from population Chelyaba 75 х АНК-17В) may be controlled by the LrSp2 gene transferred from Chelyaba 75 and linked to the gametocidal gene (Adonina et al., 2018), since the variety resisted infection, but no amplification products of markers linked to other resistance genes were detected. Lr1 gene was detected as a result of genotyping in the Omskaya 44 variety in addition to Lr19 and Lr26 identified earlier by L.V. Meshkova et al. (Meshkova et al., 2021).

Voevoda and Tulaikovskaya 10 are of interest as a source material for developing varieties with all around resistance to leaf pathogen infections under West Siberian conditions. These varieties demonstrate crop yields (509.8 for Voevoda and 405.3 g/m2 for Tulaikovskaya 10) on par with the best standard varieties. On top of that, Tulaikovskaya 10 stands out in stem length (79.9 cm), and Voevoda in high weight and number of grains per spike. Tulaikovskaya 10 was earlier used to develop the Novosibirskaya 61 spring common wheat variety, which was submitted to the FSBI “GOSSORTCOMMISSION” in 2017, but then withdrawn from testing due to lack of advantages compared to standard varieties in West Siberia branches of the FSBI “GOSSORTCOMMISSION”. In addition, including Tulaikovskaya 10 into hybridization resulted in shorter vegetation period in the lines selected from combinations with middle-late variety Sibirskaya 17 (Leonova et al., 2019). The Voevoda variety has not been involved in hybridization yet.

The analysis of the genotyping results shows that the winter varieties characterized by wheat leaf rust resistance in context of infectious background typically carry adult plant resistance genes (Lr34, Lr12, and Lr13), in particular combined with the juvenile resistance gene Lr26, whereas the spring varieties are primarily represented by donors of juvenile resistance genes, which agrees with the findings of E.I. Gultyaeva and E.L. Shaidayuk (2021). We believe that these protective mechanisms are best suited for varieties with different type of development, because there is no evidence of leaf rust infection of winter wheat varieties before the ear emergence stage in West Siberia, and therefore the transition of the pathogen from winter varieties to the spring ones appears complicated.

The results of the present study with regard to intensity of quantitative traits and crop yields of winter varieties are rather modest, because the collection samples are often characterized by low winter hardiness under local conditions, which only allows us to evaluate resistance in the context of infectious background. However, the Lr41 gene allowing the KS 93 U 62 line to resist the kLr24- clone infection despite the presence of Lr24 in the genotype was only detected in winter lines (KS 90 WGRC 10, KS 93 U 62). In addition, the Doka (with plant height of 66.5 cm and crop yield of 589.2 g/m2) and Cheshskaya 17 (80.0 cm and 547.7 g/m2) varieties may be used not only as donors for effective resistance genes (Lr26 + Lr34 and Lr9 + Lr12 + Lr13 + Lr34), but also as sources of dwarf genes not causing losses in winter hardiness and crop yields under West Siberian conditions.

## Conflict of interest

The authors declare no conflict of interest.
